# Resection and Reproduction of the Maxillæ

**Published:** 1897-10

**Authors:** G. Lenox Curtis

**Affiliations:** New York. Formerly Professor of Oral and Facial Surgery, New York Dental School and Instructor in the New York Post Graduate Medical School and Hospital.


ARTICLE V.

### RESECTION AND REPRODUCTION OF THE MAXILLÆ.

BY G. LENOX CURTIS, M. D., NEW YORK.

**Formerly Professor of Oral and Facial Surgery, New York Dental School and Instructor in the New York Post Graduate Medical School and Hospital.**

**Read before the American Medical Association at Philadelphia, June, 1897.**

The purpose of this paper is to show the profession the importance of special study and instruction in oral and facial disease, and that these are worthy of the same consideration as is given to any of the fully recognized specialities in medicine. Until they do appear in the curriculum of the medical school, the faculty will not have done its duty toward the student. The field covered by the general surgeon is altogether too great for a careful consideration of any part where such minuteness is required to save and assist nature in doing her work. The surgeon most capable of successful teaching in this line, is he who has been a thorough and conservative dentist.

Like produces like; this applies to every department in nature. The periosteum, under favorable conditions, will reproduce the substance it covered. That of the ramus of the jaw will only make the thin lamina of bone which nature has originally placed there, while that of the malar and the body of the jaw reproduces a dense structure differing materially in texture.

If the function of a part be permanently lost, reproduction is not a necessity, nature supplying only that part which is required. If the teeth have been extracted with a view to remaining out, the portion of the jaw which is required to nourish them is not reproduced, but where the teeth are replaced and retained, all, or sufficient, of the bone is reproduced and reattaches them to the jaw. Such I have seen in active use for years.

The only condition I can ascribe for the removal of the periosteum is where it is attacked by disease, such as cancer, and the entire structure destroyed. When the bone alone is destroyed, as in necrosis, cystic tumors, or from pressure by resistance of a growth, as tumor of the antrum, I see not the slightest necessity for removing this natural sheath, but on the contrary every reason for retaining it. I have seen Billroth, Von Borgmann, Agnew, Ashhurst, Garretson, and other great surgeons resect the jaw, but they invariably employed Hapfelder's Ferguson's or Landenbeck's method, except in necrosis where but small sections were involved. Liston, Tait, Barton, Mutter and Cross also followed on these lines. But what can we say for the subject? Partially or wholly jawless, maimed and disfigured for life, a repulsive and pitiable object to others and a shrinking annoyance to himself. Is it not time to call a halt and look this matter squarely in the face?

I do not censure the surgeon whose opportunities to acquire knowledge have been dwarfed by the oversight of his teachers.

My method to obtain the best results in the preservation of the contour of the jaw, is by retaining the necrosed bone in position until the periosteum has been so strengthened by the reproduction as to allow nature's outlines to be maintained, employing it as an inter-osseous splint. Where it is necessary to remove the bone, I retain the contour of the face by gauze packing and change from time to time until the bone is sufficiently reproduced to resume its shape. This requires frequent dressing so that the amount of pus may be kept at the minimum. The teeth are retained in position by means of inter-dental splints or ligatures. Where the teeth are lost, I place other teeth in the opening when the wound is nearly closed, maintaining them by artificial support and allowing the bone to form around them. Where the destruction of the bone has been great and the periosteum too weak to retain the jaw in position during the process of reproduction, I use an inter-dental splint (as employed by Liston over fifty years ago) in which the upper and lower teeth properly occlude. By hastening slowly, the danger of wounding the dental nerve is materially lessened.

I was once asked to assist a general surgeon to remove one-half of the inferior maxilla, he disclaiming it to be Sarcomotis. I saw nothing but an enlargement of the sub-maxillary gland due to the septic influence of a tooth-pulp. I labored with him to save the man's jaw and show the error he was falling into. He defiantly replied "I have said that it is a cancer of the jaw and must be taken out, and I am going to do it." And so indeed he did, thus maintaining his wisdom with the patient.

The bone he removed was as perfect as nature made it. This is but one of the many terrible examples of what results from retaining old methods.

Scarce can we read a text-book in which is not found methods on treatment of facial diseases in vogue half a century ago.

In 1886 I operated on a young woman, aged 23, with the following history. Four years previous, after suffering much pain in the face, which was swollen, a fistula appeared in the lower jaw which was diagnosed as being from an abscessed tooth. The gums around the tooth were swollen and inflamed, the molars and second bicuspid were extracted and pus continued to flow. Her health rapidly diminished, menses ceased, and had not returned although constantly under medical and surgical treatment.

Examination revealed the emaciated condition of the patient. She was suffering from blood poisoning, was highly nervous and hysterical, had no desire for food and had lost the sympathy of her doctors and family. In the left inferior maxilla where the tooth had been extracted, there were granulations. A boggy condition of the mucous membrane extended all along that side of the jaw. Over the ramus it was particularly inflamed. The probe readily passed beneath the periosteum and far up along the ramus. The patient was then too sick for an operation with a view to best results. The wound was cleansed daily to lessen the amount of pus, and for one month the patient was placed under most rigid restorative treatment with good results. I found that under the local stimulating treatment, bone had been sufficiently reproduced to strengthen the periosteum so that when I removed the dead jaw the contour was preserved. The cause of the trouble I found to be a wisdom tooth lying transversely at the neck of the jaw immediately under the condyle. This along with the granulations and debris was removed. The wound was packed continually until healthy granulations filled in the periosteum; the jaw, minus the teeth, was reproduced with all its usefulness. Complete restoration to health and a gain of twenty pounds in weight followed this work. The nerves and vessels in the jaw were not injured and no paralysis resulted.

Before my class at the New York Post Graduate Medical School and Hospital on March 25, 1893, I operated on a lad fifteen years of age who gave the following history:

Three years before, while at play he ran against a lamp post, striking the left side of his face and bruising it severely. A year later there appeared on the face, over the molar bone, a hard lump which continued to increase until it was the size of a hen's egg, preventing the boy from seeing with that eye objects on the ground near by, without bending his head. He had not realized any special pain or discomfort from the tumor. Thinking the trouble arose from the abscessed teeth, his dentist extracted the upper left first bicuspid, which showed no evidence of being diseased. I diagnosed an osseous tumor of the antrum, and found that the malar and superior maxillary bones were completely destroyed by the direct pressure against them, only the periosteum remaining. Not only was the tumor directed outward, but downward, depressing the roof of the mouth and extending beyond the alveolar process against the buccinator muscle. An incision was made through the periosteum encircling the teeth, as seen in the specimen here presented, in which the tumor and teeth are attached, and it will be noticed that only a small part of the alveolar process remained intact. This with the teeth was removed, the entire side of the face falling into the cavity made by their absence, so completely was the malar and the superior maxillary bones destroyed. The inferior orbital ridge and zygoma only resisting the pressure of the tumor. A profuse hemorrhage followed its removal, but was readily checked by hot water. The wound was packed with aristol and gauze, and the contour of the face secured. The periosteum united with antures. Through this opening the wound was dressed until the shape of the face was permanently restored. Time of operation twenty-five minutes.

The following day there was considerable œdema which readily subsided. From day to day the dressing was changed until the periosteum could support itself, and in two weeks the case was dismissed from the hospital The antrum was douched daily until restoration was complete. An artificial denture was made to replace those lost, to give the normal fullness to the mouth. In this operation there was no external wound, consequently no necessity for ligature and no scarring of the face which would necessarily follow had the operation been done on the lines drawn in general surgery. The wound completely healed in six weeks with no deformity of the face. I have seen the case from time to time and in every way it is eminently satisfactory.

On Feb. 3, '93, I operated on a gentleman 73 years of age who, up to the year prior to then, was in robust health never needing a physician in forty years. He stated that he applied to a dentist to have the left superior wisdom tooth, which was loose, removed, it having elongated, owing to the loss of its antagonist. As the dentist was using the forceps, the patient noticed they were covered with blood, but before he could rebel against this outrage, a tooth had been extracted, which was found firmly attached and resistent. He saw that a sound and healthy tooth had been taken out by mistake. The dentist then removed the loose tooth with slight inconvenience. The wound made by the extraction of the teeth did not heal, and the gums around it became swollen and inflamed, the remaining teeth on that side soon were loose and sore. In three weeks they were so troublesome that with his fingers he removed the first molar. He then noticed an opening into the antrum and that granulations protruded.

About two months after, the bicuspid was extracted in a similar manner, and in two weeks later the cuspid. The entire side of the jaw became very painful; the patient was unable to sleep or take proper nourishment and rapidly diminished in strength and health, until at this time, Feb. 3, he was extremely emaciated, not having taken solid food for weeks and for the past three days only water, because of the great pain in the effort to swallow.

Examination revealed a deplorable condition of affairs; the entire left half of the jaw and cheek were infiltrated. The microscope showed epithelioma. The characteristic cancerous odor prevailed. An incision was made anterior to the right cuspid and extended back and across the soft palate to the condyle, down the ramus, and forward along the buccal surface of the jaw to cuspid upward and forward, until all the mucous membrane to the median line was removed. The entire enclosed area was then resected, leaving only the external portion of the malar bone and the orbital ridge of the superior maxillary in position. In operating, I removed a portion of the anterior lobe of the parotid gland, along with the duct. The hemorrhage was profuse, but was completely checked in a minute by hot water. The wound was dried, packed with aristol and gauze, no ligatures being employed. Time of operation, twenty minutes. The patient made a splendid recovery, slept comfortably much of that night and had but slight rise of temperature. There was some œdema of the face which lasted three days, Patient received most nourishing diet and was sitting up in three days. On the seventh day following the operation, the case was for the first time dressed, no untoward symptoms arising in the meantime. The packing was perfectly dried and scarcely blood stained; not a drop of pus was present. The wound was repacked, but loosely, and redressed every third day without a single complication. On Feb. 17th, patient was dismissed from the hospital with instruction to douche the wound frequently. The wound made rapid progress in healing, and on the thirteenth day of March, it had almost completely closed, leaving but a single opening into the antrum, so that on the 30th day of March the impression for an artificial denture was taken.

The patient's health had wonderfully improved, he being free from pain and slept soundly. He was able to resume the management of his affairs and was again in good health, continuing so until August, '94, when he contracted pneumonia from which he died. Two months prior to his death, the cancerous granulations appeared in the old wound. There was no disfigurement of the face from the operation, and the only inconvenience was the loss of his natural teeth, as he was unable to wear the artificial substitute.

A lad 13 years of age was brought to me in March, 1893. Giving a history of complete nasal stenosis of some years' standing, a clear history of adenoids. He had been under medical care for years, the physicians failing to recognize the cause of his trouble. The discharge of pus from the mouth and nose caused the physician to refer the patient to a general surgeon, who in turn referred the case to me, with the statement to the parents that he believed the preservation of the jaws was preferable to their removal which would result in a hideous disfigurement from an operation at his hands. I have never operated on a more lifeless and waxy looking creature. The odor from his breath and body, which was steeped in pus, was most sickening. There was no time to lose. Desperate chances had to be taken to save his life. All of the upper oral and bicuspid teeth were so loose that but for the periosteal attachment they would have dropped out. There were large sinuses under the lips and the roof of the mouth through which pus exuded. The periosteum of the roof of the mouth was so filled with pus that it bagged. All of the bone of the superior maxillæ, anterior to the first molar including the palatal plate was necrosed, likewise the palatal bones and the inferior turbinates. The adenoids completely filled the nares and crowded into the antrum of Highmore, breaking down the walls, and advanced until the process of destruction was complete.

Pus oozed from all the loose teeth and through the sinuses, nose, mouth and out up through the lachrymal ducts into the eyes. The throat was so plugged up that breathing was very difficult and ptyglism extreme. At this operation I removed what I could of the adenoids, opening the nasal passage and partially cleaning the antrums, which was done with great difficulty; as the boy took chloroform badly, owing to extreme anæmia; the loss of blood was not so very great.

The loose teeth were supported by ligatures until held by the new bone.

The patient made very good recovery from the operation, and in forty-eight hours the improvement was noticeable. The wounds were dressed daily and through the sinuses douched every hour. Recovery was so rapid that in four weeks I was again able to operate, this time removing all of the necrosed bone and thoroughly curetting the antrums.

The boy made rapid strides toward recovery, and by May 1st I was able to perform the final operation, when I removed the remaining adenoids. Here occurred a profuse hemorrhage, but with per-oxide and hot water it was quickly checked. Most stimulating tonics and nourishing diet were prescribed from this on. Recovery was marvellous, so that in two weeks the patient was able to walk to my office for treatment. By June 1st, he was dismissed cured, having gained 40 lbs. in weight, having regained his normal color, good appetite and usual strength. He then went to his country home. I saw him again in one year. There was no occasion for treatment, with the exception of a slight imperfection in the alveolar process, where there had been a deep sinus, the gums did not unite and a few adenoids had put in an appearance. The only additional opening through which I operated, was made where I extracted the left superior molar, this being so badly diseased I considered it well out, and through which I was better able to reach the antrum.

To facilitate cleansing of the wound and destruction of the pus, let me recommend to you Electrozone, the best of all agents I have found for this purpose. Under its use the pus melts away like the dew before the morning sun.

I have done many cases similar to these stated and without deformity in any instance. All of them are accompanied by blood-poisoning and have usually been treated for rheumatism, malaria and typhoid fever for months and even years, before the error is discovered.

The conservative method is not conducive to a fine collection of pathological specimens, as a recovery of a part without blemish leaves only the history of the case and the statement of the patient as proof of the malady.

The student should have a chance to see in practice the methods he wishes to adopt. In this city, only recently at our great University no end of strife resulted from a determined and successful effort of the dental department to make a place in the hospital for their oral surgeon, with equal right to operate. This was the beginning of the end when all such institutions must of necessity adopt the same course. Why confine this work to so-called major operations when they can be simplified to the minor class by annexing such men skilled in dentistry and medicine alike to the medical faculty?

Let us hope that the controllers of such faculties will appreciate that "Knowledge is power," and that new methods are a necessity.—*Dominion Dental Journal.*

